# ELL2 Is Downregulated and Associated with Galactose-Deficient IgA1 in IgA Nephropathy

**DOI:** 10.1155/2019/2407067

**Published:** 2019-06-04

**Authors:** Youxia Liu, Jie Zheng, Na Zhao, Junya Jia, Tiekun Yan

**Affiliations:** ^1^Department of Nephrology, Tianjin Medical University General Hospital, Tianjin, China; ^2^Radiology Department, Tianjin Medical University General Hospital, Tianjin, China; ^3^Department of Nephrology, Qianfoshan Attached Hospital of Shandong University, Jinan, China

## Abstract

**Background:**

Galactose-deficient IgA1 (Gd-IgA1) is an important causal factor in IgA nephropathy; however, the underlying mechanism for the production of Gd-IgA1 is unknown. The elongation factor for RNA polymerase II (ELL2), which encoded a key component of the superelongation complex (SEC), drives secretory-specific Ig mRNA production.

**Methods:**

We enrolled 21 patients with IgAN, 18 healthy controls, and 20 patients with non-IgAN glomerulonephritis. The differential expression of ELL2 was compared using publically available data from Gene Expression Omnibus (GEO) datasets. The relationship between ELL2 expressions and galactose-deficient IgA1 (Gd-IgA1) levels in serum were also studied. At last, the results were validated by shELL2 treatment experiment.

**Results:**

We found that the number of CD19+ B cells was increased in IgAN patients compared to healthy controls. The expression level of ELL2 in patients with IgAN was significantly lower than that of healthy control and disease control. Consistent with present results, the lower ELL2 expression in IgAN patients was observed in microarray expression profiles from GEO datasets. Pearson correlation analysis showed that ELL2 expression negatively correlated with Gd-IgA1 levels. Furthermore, in an in vitro experiment, we found that loss of ELL2 function in human B lymphoma DAKIKI cells, an IgA1-producing cell line, increased the levels of Gd-IgA1, which confirmed that ELL2 modulated the levels of Gd-IgA1.

**Conclusion:**

Our findings implied that decreased ELL2 expression was negatively correlated with the numbers of B cells and aberrant glycosylation of IgA1 in IgAN.

## 1. Introduction

Immunoglobulin A nephropathy (IgAN) is the most common glomerulonephritis in the world, and approximately 40% of patients progress to end-stage kidney disease (ESKD) within 20 years after renal biopsy [1, 2]. Recent studies indicated that galactose-deficient IgA1 (Gd-IgA1) as the trigger factor can form large immune complexes with anti-glycan IgG antibodies [3, 4]. These complexes bind to glomerular mesangial cells resulting in stimulation of cell proliferation, activation of complement, and release of inflammatory mediators which ultimately leads to occurrence of IgAN [5, 6]. Researchers have reported that high levels of Gd-IgA1 were not only associated with IgAN pathogenesis but with disease progression [7]. Therefore, delineating the abnormal production of Gd-IgA1 is important in understanding IgAN. Many studies found a genetic association between C1GALT1, ST6GALNAC2, and Cosmc genes and IgAN; however, there is a discrepancy of information on the expression of these glycosyltransferases [8–10]. Two genome-wide association studies (GWAS) conducted in IgAN populations identified C1GALT1 and/or C1GALT1C1 genes that strongly associated with levels of Gd-IgA1, however, which only explains approximately 2%-7% of the variability in Gd-IgA1 levels, suggesting that other factors are important in determining Gd-IgA1 levels in individuals [11, 12].

The elongation factor for RNA polymerase II (ELL2), a novel mucin protein, encodes a key component of the superelongation complex (SEC) that drives secretory-specific Ig mRNA production [13, 14]. A genome-wide association study conducted in multiple myeloma discovered that ELL2 mutation is associated with total IgA levels [15]. More recently, Kiryluk et al. found an association of Gd-IgA1 with *ELL2* (rs56219066, *P* = 8.5 × 10^−3^) [12]. In addition to influencing Ig production, ELL2 could influence the survival and proliferation of B-cells [13, 16]. These studies suggested the potential involvement of ELL2 in Gd-IgA production and the pathogenesis of IgAN.

Therefore, the aim of this study was to evaluate whether the expression of ELL2 in B lymphocytes was associated with Gd-IgA1 levels in patients with IgAN, as well as its possible underlying mechanism of ELL2 in the glycosylation of IgA1 molecules.

## 2. Materials and Methods

### 2.1. Subjects

A total of twenty-one IgAN patients diagnosed in Tianjin Medical University General Hospital from May to August 2017 were enrolled in this study. The diagnosis was based on the deposition of IgA in the glomerular mesangium by immunofluorescence detection, as well as the lack of clinical or serological evidence of other inflammatory conditions, such as Henoch-Schonlein purpura. At the same time, 18 healthy volunteers whose age and gender matched with patients were recruited. 13 participants from each group were used for CD19+ cell analysis by flow cytometry.

Twenty patients with non-IgAN glomerulonephritis, including lupus nephritis (*n* = 5), ANCA-associated vasculitis (*n* = 5), minimal change disease (*n* = 5), and membranous nephropathy (*n* = 5) were selected as disease controls. Plasma was collected from all individuals in this study, for patients at the time of renal biopsy. The plasma samples were stored in aliquots at -80°C for the subsequent use. Clinical information, including 24-hour urine protein excretion, blood pressure, and total IgA levels, were collected at the time of renal biopsy. The estimated glomerular filtration rate (eGFR) was calculated using the Chronic Kidney Disease Epidemiology Collaboration creatinine equation. The histological lesions were classified according to the Oxford classification system. The Medical Ethics Committee of Tianjin Medical University General Hospital approved the study protocol, and informed written consent was obtained from all individuals.

### 2.2. Assay for IgA1 and Gd-IgA1

Total IgA1 and Gd-IgA1 levels in plasma and in cell culture supernatant were determined by ELISA, as previously reported [7]. A standard consisting of native IgA1 purified by normal human plasma (EMD Chemicals, USA) was used as the standard for the quantification of total IgA1. As for Gd-IgA1 detection, the standard was used which consists of IgA1 protein isolated from plasma of a patient with multiple myeloma using an agarose-bound jacalin affinity chromatography column (Pierce Chemical Company, USA). The IgG was removed by a protein G column (GE, USA). The terminal sialic acid from O-linked GalNAc on bound samples and the standard IgA1 protein were removed by neuraminidase (Roche, USA), and galactose from O-linked GalNAc was removed by galactosidase (Sigma, USA). As the standard IgA1 protein is not entirely devoid of galactose, we expressed the results as U/mL, in which 1 unit of Gd-IgA1 was defined as 1 ng of this standard Gd-IgA1 protein.

### 2.3. B Lymphocyte Isolation

About 5 mL venous blood sample was taken into ethylenediaminetetraacetic acid- (EDTA-) anticoagulated tubes. Peripheral blood mononuclear cells (PBMCs) were separated by density-gradient centrifugation on Ficoll (TBD, China), then washed three times with phosphate-buffered saline (PBS) and resuspended in PBS + 1% bovine serum albumin (BSA). Peripheral B lymphocytes were isolated using anti-human CD19 positive magnetic beads (Miltenyi Biotec, 130-050-301, USA) according to the manufacturer's instructions.

### 2.4. Flow Cytometry

The cell flow cytometry assay was performed using FITC-conjugated, anti-CD19 mAb (BioLegend, 392508, USA) according to the manufacturer's instructions.

### 2.5. RNA Extraction and Real-Time Reverse Transcription PCR (RT-PCR)

Total cellular RNA was extracted from CD19-positive B lymphocytes using the TRIzol Reagent (Invitrogen, USA). RNA quantity was determined using the NanoDrop ND-1000 spectrophotometer. cDNA was synthesized using 300 ng total RNA with the reversed first-strand cDNA kit according to the manufacturer's protocol (Promega, USA). Resulting cDNA was amplified with a 20 *μ*L reaction mixture using SYBR Green PCR Master Mix (Roche, USA) in an Applied Biosystems 7500 Real-Time PCR System. Levels of ELL2 transcripts were expressed relative to the expression of the *GAPDH* housekeeping gene. The results were expressed in terms as a relative quantity to the calibration curve. Relative expression was calculated using the 2^-△△CT^ method. The primer pairs of ELL2 and GAPDH are listed in Table 1. GAPDH gene amplification was used as a reference standard to normalize the target signal.

### 2.6. DAKIKI Cell Culture and shRNA Knockdown Studies

The DAKIKI cell line was purchased from ATCC (Manassas, USA) and cultured in RPMI 1640 medium containing 10% heat-inactivated fetal calf serum in a humidified atmosphere at 37°C with 5% CO_2_. The cell line was authenticated by short tandem repeat profiling and tested for mycoplasma contamination. The short-hairpin RNA (shRNA) targeting ELL2 and shRNA negative control were purchased from Jikai Genechem (China). ShRNA targeting sequences of ELL2 was CTGCAAATACAATTCGAAA. DAKIKI cells in the logarithmic growth phase (1 × 10^5^/well) were seeded in a 24-well plate. These cells were transfected with the GFP-expressing lentiviral vector shControl or shELL2, and 10 ng/mL polybrene (Jikai Genechem, China) was added. After culture for 24 h, the medium was refreshed. Fluorescence was detected after 72 h of incubation using the fluorescence microscope. The lentiviral shELL2-transfected DAKIKI cells were screened with puromycin (Sigma-Aldrich, USA), and successful transfectants were used for subsequent experiments.

### 2.7. Western Blot Analysis

The amount of ELL2 and *β*-actin was determined by Western blotting analysis. Total protein extracts were prepared with lysis buffer, which was separated on 10% SDS-PAGE and then transferred to the polyvinylidene difluoride membrane (Millipore). The membranes were incubated in 5% nonfat milk powder diluted in PBS containing 0.1% Tween-20 for 2 hours at room temperature and probed with rabbit polyclonal anti-ELL2 antibody (Abcam, ab219865, USA) and anti *β*-actin monoclonal antibody (Sigma, Japan) in blocking buffer overnight at 4°C. Finally, membranes were incubated with a secondary antibody of horseradish peroxidase-conjugated goat anti-rabbit IgG (Bio-Rad, USA). Immunocomplexes were detected with the enhanced chemiluminescence method (GE Healthcare, USA).

### 2.8. Bioinformatic Analysis

The differential expressions of suspected IgAN candidate genes were compared with those of healthy controls using publically available data from Gene Expression Omnibus (GEO) using “IgA nephropathy” as the search term. Three experiments (GSE73953, GSE58539, and GSE14795) conducted in whole-blood samples were included in the current analysis [17–19]. Through the PreProcessCore package of R, the raw data was normalized and differential expression genes (DEGs) between IgAN patients and controls were screened out based on the limma package with the criteria of |log FC| > 1 and *P* value <0.05.

### 2.9. Statistical Analysis

For continuous variables, data with a normal distribution was expressed as mean ± SD and compared by an independent-samples *t* test, whereas other data were expressed as the median (first quartile and third quartile) and analyzed by the Mann–Whitney *U* test. Receiver operating characteristic (ROC) curves were used to judge the diagnostic utility of ELL2 levels. For ROC curves, the best cutoff values were chosen according to the highest diagnostic accuracy determined using the Youden index: sensitivity - (1 - specificity). The level of significance was chosen as *P* < 0.05. All statistical tests were performed using SPSS version 16.0.

## 3. Results

### 3.1. Baseline Clinical Characteristics of Patients with IgAN

The clinical and pathological features of IgAN patients in the present study are shown in Table 2. There are 11 males and 10 females with an average age of 39 years. The median of proteinuria is 1.18 g/d, and the mean eGFR is 82.81 mL/min/1.73 m^2^ of IgAN patients on biopsy. The level of IgA1 (median: 1810 *μ*g/mL, interquartile range 1445-2153 *μ*g/mL) in IgAN patients is significantly higher compared to that of the healthy controls (1450 *μ*g/mL, 840-1758 *μ*g/mL, *P* = 0.04). The grading of pathological lesions by Oxford classification is shown in Table 2.

### 3.2. Patients with IgAN Had a High Number of CD19+ B Cells and a Low Expression Level of ELL2

**Figure 1:**
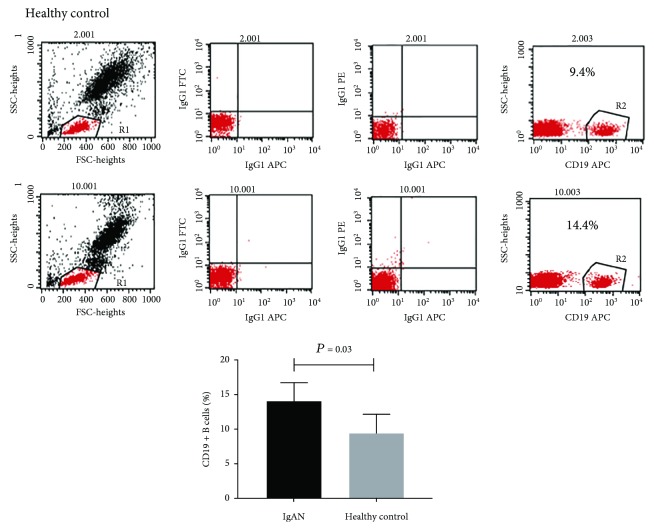
Flow cytometry analysis of CD19+ cells in blood of 13 IgAN patients and 13 healthy controls. Peripheral blood mononuclear cells (PBMCs) of donors were prepared by Ficoll-Paque density centrifugation and subjected to flow cytometry analysis. Representative gating strategy for enumeration of total CD19+ cells in peripheral blood. The percentage of CD19+ B cells in PBMCs was increased in IgAN patients compared to healthy controls (*P* = 0.03).

**Figure 2:**
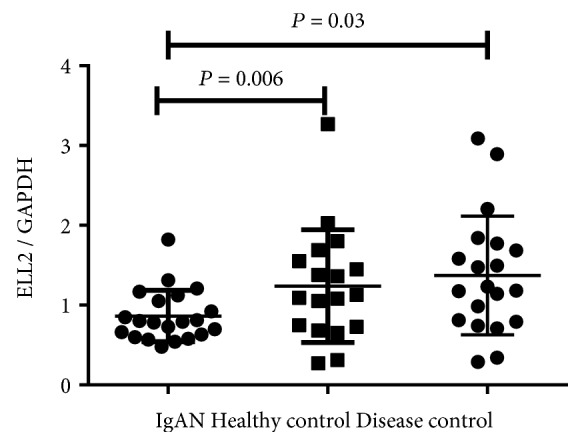
The mRNA expression levels of ELL2 evaluated by real-time PCR in B cells in 21 patients with IgAN, 18 healthy controls, and 20 disease controls. ELL2 mRNA levels were significantly lower in patients with IgAN than in healthy subjects and in those disease controls (including 5 patients with lupus nephritis, 5 with ANCA-associated vasculitis, 5 with minimal change disease, and 5 with membranous nephropathy), as shown by RT-PCR. ELL2 expression levels were normalized on the housekeeping gene GAPDH. The scatter plots represent mean ± SEM.

**Figure 3:**
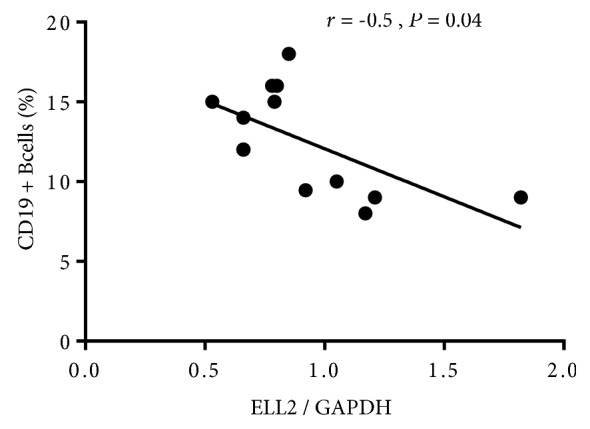
The correlation between ELL2 mRNA levels in B cells and B cell numbers in 13 patients with IgAN. ELL2 mRNA levels inversely correlated with B cell numbers (*r* = −0.5, *P* = 0.04).

In circulation, CD19+ B lymphocytes are the major cells for IgA1 production. Blood CD19+ B cells were analyzed by flow cytometry. As shown in Figure 1, the number of CD19+ B cells was increased in IgAN patients compared to healthy controls (*P* = 0.03). We next detected the different expression of the ELL2 mRNA level in B cells. The result showed that the ELL2 mRNA level of patients with IgAN was significantly lower than that of the healthy control (0.86 ± 0.31 vs. 1.37 ± 0.75, *P* = 0.006) and disease control (0.86 ± 0.31 vs. 1.23 ± 0.71, *P* = 0.03, Figure 2). The level of ELL2 mRNA was negatively related with the number of CD19+ cells in IgAN (*r* = −0.5, *P* = 0.04, Figure 3).

### 3.3. Differential Gene Expression Analysis Suggests ELL2 Involvement in IgAN Pathogenesis

Gene expression profiling has provided great insight into IgAN pathogenesis. We ascertained whether the associated ELL2 gene was expressed differently in patients with IgAN and healthy controls. Consistent with the present results, we noticed that downregulated mRNA expression of ELL2 in IgAN patients was observed in PBMCs (GSE73953, *P* < 0.001) and CD14+ cells (GSE58539, *P* = 0.02). For GSE 14759, the ELL2 mRNA level in IgAN was also lower than that of control; however, the difference was not significant (*P* = 0.09) (Table 3).

### 3.4. Cutoff Values of ELL2 for Differential Diagnosis of IgAN

Next, the cutoff values of ELL2 for differential diagnosis of IgAN were obtained from ROC analysis. The optimum ELL2 cutoff point for predicting IgAN was defined as 0.95 with a sensitivity of 71% and a specificity of 68%.

### 3.5. Expression of ELL2 Related with the Gd-IgA1 Levels

**Figure 4:**
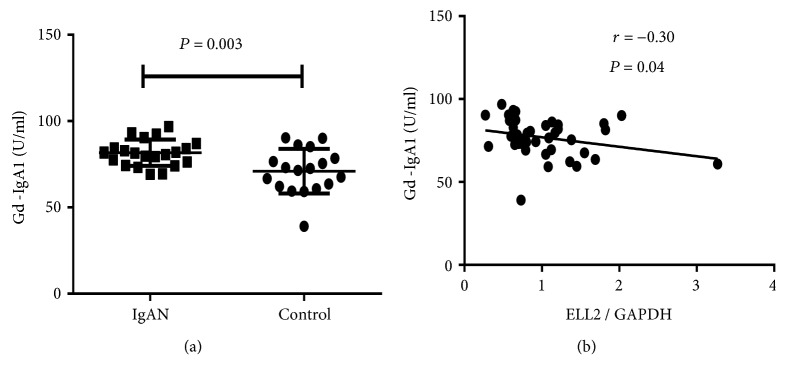
Gd-IgA1 levels in plasma (a) and the correlation between ELL2 and Gd-IgA1 levels (b) in 21 patients with IgAN and 18 healthy controls. (a) ELL2 expression levels were significantly lower in patients with IgAN than in healthy controls. ELL2 expression levels were normalized on the housekeeping gene GADPH. The scatter plots represent mean ± SEM. (b) ELL2 mRNA levels inversely correlated with Gd-IgA1 levels (*r* = −0.3, *P* = 0.04).

First, we evaluated Gd-IgA1 levels in patients with IgAN and healthy control. As shown in Figure 4(a), patients with IgAN had significantly higher Gd-IgA1 levels (81.46 U/mL, interquartile range 75.31-87.19 U/mL) compared with healthy control (72.00 U/mL, 61.79-80.12 U/mL, *P* = 0.003). After the identification that IgAN patients had a relatively low level of ELL2 mRNA and high levels of Gd-IgA1, we next investigated whether ELL2 expression correlated with Gd-IgA1 levels. ELL2 expression negatively correlated with Gd-IgA1 levels (*r* = −0.30, *P* = 0.04, Figure 4(b)). The negative correlation between ELL2 mRNA and Gd-IgA1 suggested that lower ELL2 expression might be involved in the mechanism of increased Gd-IgA1 production in IgAN.

### 3.6. ELL2 Knockdown Experiments Confirm Involvement of ELL2 in Gd-IgA1 Production

**Figure 5:**
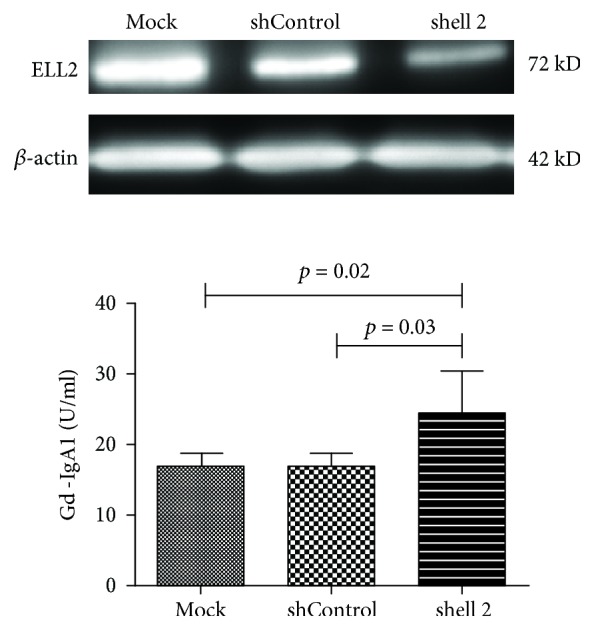
Gd-IgA1 production after knockdown ELL2 in DAKIKI cells. Western blot of ELL2 confirmed robust knockdown. Transfection of DAKIKI cells with shELL2 resulted in reduction in ELL2 protein expression. A significant increase in Gd-IgA1 levels was shown in DAKIKI cells transfected with shELL2 treatment. “Mock” indicates mock-transfected cells going through the transfection processes without addition of shRNA.

To confirm the direct cause-and-effect relationship between ELL2 expression and Gd-IgA production, DAKIKI cells were transfected with shRNA targeting ELL2 (shELL2) to silence the activity of ELL2. Next, we evaluated changes in gal-deficient IgA1 levels. Consistent with the observed in vivo effect, in vitro knockdown of ELL2 expression resulted in a remarkable increase in Gd-IgA1 levels in DAKIKI cells (*P* = 0.03 for mock and *P* = 0.02 for shControl, Figure 5).

## 4. Discussion

Increasing evidence suggests that aberrant O-glycosylation of IgA1 acts as a “trigger” in the pathogenesis of IgAN. ELL2 is a member of an ELL family of RNA polymerase II elongation factors that regulates B lymphocyte proliferation and IgA production [20]. In the current study, we first validated the decreased ELL2 expression level in B lymphocyte in patients with IgAN inducing the increased production of aberrantly glycosylated IgA1 molecules, which ultimately contributed to IgAN pathogenesis.

The mechanisms leading to aberrant glycosylation of IgA1 have been studied extensively; so far, the identified genes associated with levels of Gd-IgA1 only explain less than 10% of the variability in Gd-IgA1 levels, suggesting we need to look for other factors determining Gd-IgA1 levels in individuals. Previous evidences showed that ELL2 was deregulated expressed in prostate cancer and myeloma [21–23]. It is reported that knockdown of ELL2 enhanced cell proliferation, migration, and invasion. Park et al. observed that the number of immature and recirculating B cells in the bone marrow was increased in ELL2 deletion mice, which suggested that ELL2 loss could enhance B cell proliferation [13]. The percentage of circulating B lymphocytes was increased in IgAN patients with increased concentration of IgA in serum [24]. Animal studies indicated that inhibition of B cell death caused the development of IgAN in transgenic mice [25]. Recent potential targets for therapy in IgAN have focused on the B-cell activation factor of the TNF family (BAFF) and A proliferation-inducing ligand (APRIL), which promotes B lymphocyte proliferation and IgA class switching [26, 27]. In the present study, we found that CD19+ cell number was significantly increased in IgAN, which negatively correlated with ELL2 expression. Taken together, these data suggested that ELL2 acted an important role in B cell proliferation in IgAN patients.

In the present study, we observed downregulated ELL2 mRNA expression in B lymphocytes in IgAN patients. To directly inform of a possible relationship between the ELL2 and Gd-IgA1 levels, we exploited the fact that samples with lower ELL2 expression levels showed higher levels of secreted IgA1 and Gd-IgA1 in vivo. Furthermore, the role of ELL2 in IgA1 O-glycosylation regulation was also confirmed by transfection experiments in DAKIKI cells in vitro showing that ELL2 modulated the levels of circulating aberrant glycosylated IgA1. These findings indicated again that ELL2 might be involved in the pathogenesis of IgAN as a causal factor for promoting the production of Gd-IgA1. Recently, a GWAS from Scotland which comprised 1096 individuals showed that variation in ELL2 influenced the genetic control of IgG glycosylation [20]. The multiple myeloma risk allele confers lower ELL2 expression, which is associated with reduced levels of IgA and IgG [23, 28]. Ali et al. speculated that one possibility is that lower Ig levels could lead to slower antigen clearance and stimulation of the B cell system for longer periods of time, and hence, a higher likelihood of malignant transformation [23]. Previous studies have shown that IgAN susceptibility is associated with risk alleles being involved in immune defense. These findings suggested that, similar to the role played in multiple myeloma, reduced ELL2 expression may act an important role in B cell proliferation and make the production of secreted Ig less efficient, which resulted in a higher likelihood of a damaging immunologic response to Gd-IgA1 in the circulation and thereby a higher risk of IgAN. The human study presented herein provides the first evidence of ELL2's participation in IgAN pathogenesis and suggests the potential mechanism related to production of Gd-IgA1.

The small sample size was the major limitation of this study. Further studies with larger sample sizes are needed to assess the target mRNA relationship with serum galactose-deficient IgA1 level in IgAN. Furthermore, we only performed these experiments in DAKIKI cells; more than one cell line should be used in further studies to validate these associations.

## 5. Conclusion

In summary, our findings implied that decreased ELL2 expression was negatively correlated with the number of B cells and aberrant glycosylation of IgA1 in IgAN. Future studies were needed to verify in more patients ELL2 as a causal factor for promoting the production of Gd-IgA1 in IgAN.

## Figures and Tables

**Table 1:** Primers used to amplify the *ELL2* and *GAPDH* genes.

Gene	Forward primers (5′-3′)	Reverse primers (5′-3′)
*ELL2*	CATCACCGTACTGCATGTGAA	ACTGGATTGAAGGTCGAAAAGG
*GAPDH*	TTGCCCTCAACGACCACTTT	TGGTCCAGGGGTCTTACTCC

**Table 2:** The baseline data for patients with IgAN and healthy controls.

Characters	Mean ± SD or *n* (%)		
	IgAN	Healthy controls	*P*
Male/female	11/10	9/9	
Age (mean ± SD, year)	39 ± 12	37 ± 13	0.84
SBP (mmHg)	125 ± 18	126 ± 16	0.67
Proteinuria (g/d, median, IQR)	1.18 (0.48-3.39)		
Plasma IgA1 (*μ*g/mL, median, IQR)	1810 (1445-2153)	1450 (840-1758)	0.04
eGFR (mL/min/1.73/m^2^)	82.81 ± 25.78		
Oxford classification (%)			
M score (M0/M1)	4 (19)/17 (81)		
E score (E0/E1)	12 (57.1)/9 (42.9)		
S score (S0/S1)	11 (52.4)/10 (47.6)		
T score (T0/T1/T2)	9 (42.8)/9 (42.8)/3 (14.4)		
C score (C0/C1/C2)	5 (23.8)/9 (42.8)/7 (33.4)		

SD: standard deviation; SBP: systolic blood pressure; eGFR: estimated glomerular filtration rate.

**Table 3:** ELL2 mRNA expressions in patients with IgA nephropathy compared with healthy controls from GEO database.

Gene	Experiment E-GEOD-73953			Experiment E-GEOD-58539			Experiment E-GEOD-14795		
	IgAN (*n* = 15)	Control (*n* = 2)	*P* value	IgAN (*n* = 8)	Control (*n* = 9)	*P* value	IgAN (*n* = 12)	Control (*n* = 8)	*P* value
*ELL2*	87.7 ± 57.1	377.6 ± 100.1	<0.001	0.01 ± 0.02	0.03 ± 0.04	0.02	128.3 ± 71.3	185.4 ± 69.6	0.09

Data are means ± SD. IgAN: IgA nephropathy.

## Data Availability

Raw data used during the current study are available from the corresponding author on reasonable request for noncommercial use.

## Abbreviations

IgAN: Immunoglobulin A nephropathy
ESKD: End-stage kidney disease
Gd-IgA1: Galactose-deficient IgA1
GWAS: Genome-wide association study
ELL2: Elongation factor for RNA polymerase II
eGFR: Estimated glomerular filtration rate
RT-PCR: Reverse transcription PCR
GEO: Gene expression omnibus.
